# Beyond chaperoning: HSPA8 modulates alternative splicing of PPP2R2D to drive apoptotic evasion and macrophage-associated immune microenvironment remodeling in triple-negative breast cancer

**DOI:** 10.3389/fonc.2026.1941438

**Published:** 2026-10-05

**Authors:** Wenting Xu, Qingwen Zheng, Yu Si, Tian Yao, Hao Lei, Yongtao Li

**Affiliations:** Breast Surgery Department, Affiliated Cancer Hospital of Xinjiang Medical University, Urumqi, Xinjiang, China

**Keywords:** alternative splicing, HSPA8, M2 macrophage polarization, PPP2R2D, triple-negative breast cancer

## Abstract

**Introduction:**

The molecular chaperone HSPA8 is classically recognized for its role in protein homeostasis, and while its upregulation correlates with poor prognosis in triple-negative breast cancer (TNBC), whether HSPA8 exerts non-canonical functions beyond chaperoning remains unknown.

**Methods:**

Through integrative transcriptomic profiling, RNA immunoprecipitation (RIP), isoform-specific rescue experiments, and *in vivo* xenograft models, we investigated the functional and mechanistic role of HSPA8 in TNBC pathogenesis.

**Results:**

We unveil a paradigm-shifting function for HSPA8 as a modulator of alternative splicing of the PP2A regulatory subunit gene PPP2R2D, promoting exon 2 retention to favor the full-length PPP2R2D-2 isoform over the truncated PPP2R2D-1. These two isoforms exhibit functionally distinct roles: only PPP2R2D-2 possesses pro-survival activity, whereas PPP2R2D-1 lacks this function. Specifically, PPP2R2D-2 rescues HSPA8 depletion-induced apoptosis by modulating the mitochondrial pathway (BAX/BCL-2/cleaved Caspase-3) and restores *in vivo* tumor growth. Strikingly, this splicing axis also correlates with an immunosuppressive tumor microenvironment, characterized by altered M1/M2 macrophage marker abundance.

**Discussion:**

These findings uncover a dual mechanism that simultaneously drives intrinsic survival signaling and is associated with alterations in the tumor immune microenvironment. They transcend the classical chaperone paradigm by establishing HSPA8 as a non-canonical splicing modulator, and the HSPA8-PPP2R2D-2 axis emerges as a critical vulnerability in TNBC, offering a novel therapeutic angle that concurrently targets tumor cell survival and microenvironmental immunosuppression.

## 1 Introduction

Triple-negative breast cancer (TNBC), defined by the absence of estrogen receptor, progesterone receptor, and HER2 expression, accounts for 15-20% of all breast cancer cases and is characterized by high heterogeneity, aggressive clinical behavior, and poor prognosis (1, 2). Unlike other breast cancer subtypes, TNBC lacks effective molecular targets, leaving chemotherapy as the primary systemic treatment option. However, chemoresistance and subsequent tumor recurrence remain major clinical challenges (3). Apoptotic evasion is increasingly recognized as a fundamental mechanism driving TNBC progression and treatment resistance (4), yet the molecular underpinnings of this phenotype remain incompletely understood.

Alternative splicing (AS) is a critical post-transcriptional mechanism that expands proteomic diversity by enabling a single gene to produce multiple mRNA and protein isoforms (5). Dysregulated AS is now considered a hallmark of cancer, contributing to tumor initiation, progression, and therapeutic resistance (6, 7). In TNBC, aberrant splicing events have been shown to promote malignant phenotypes including proliferation, invasion, metastasis, and drug resistance (8). Splicing factors such as SNRPB2 and CPSF4 have recently been implicated in TNBC progression through regulating MDM4 and HMG20B splicing, respectively (9, 10). Nevertheless, the upstream regulatory networks governing AS dysregulation in TNBC remain largely unexplored.

HSPA8 (also known as heat shock cognate 70, HSC70) is a constitutively expressed member of the HSP70 family traditionally recognized for its chaperone functions in protein folding, transport, and degradation (11). Accumulating evidence indicates that HSPA8 is involved in the progression of various malignancies, with studies suggesting it may exert oncogenic functions in certain cancer contexts (12, 13). We previously reported that high HSPA8 expression correlates with poor prognosis in TNBC patients through bioinformatic analysis and clinical sample validation (14). However, whether HSPA8 possesses non-canonical functions beyond protein homeostasis—particularly in RNA metabolism and splicing regulation—remains unknown.

PPP2R2D encodes a regulatory subunit of protein phosphatase 2A (PP2A), a major tumor suppressor implicated in signal transduction, cell cycle control, and apoptosis (15, 16). Through database mining, we identified that PPP2R2D undergoes alternative splicing to generate two major isoforms: the full-length PPP2R2D-2 (including exon 2) and the truncated PPP2R2D-1 (resulting from exon 2 skipping). Whether these isoforms possess distinct functions and how their balance is regulated in TNBC have not been investigated.

To identify potential downstream targets of HSPA8, we performed RNA-sequencing followed by rMATS analysis in HSPA8-knockdown TNBC cells. Among the differentially spliced genes, PPP2R2D emerged as a significant hit, with HSPA8 depletion markedly altering its exon 2 inclusion/skipping ratio. PPP2R2D encodes a regulatory subunit of protein phosphatase 2A (PP2A), a major tumor suppressor implicated in signal transduction, cell cycle control, and apoptosis (15) (16). Notably, PPP2R2D has been reported to be dysregulated in gastric cancer and associated with advanced tumor stage and poor prognosis (17). However, whether PPP2R2D undergoes alternative splicing and how this process is regulated in TNBC remain unknown. Based on these observations, we hypothesized that HSPA8 may regulate PPP2R2D alternative splicing to promote TNBC progression.

In this study, we demonstrate that HSPA8 is physically associated with PPP2R2D pre-mRNA within a complex and modulates its alternative splicing, favoring the production of the full-length PPP2R2D-2 isoform. PPP2R2D-2, but not PPP2R2D-1, mediates the anti-apoptotic function of HSPA8 by modulating the mitochondrial apoptosis pathway (BAX/BCL-2/cleaved Caspase-3). Moreover, the HSPA8- PPP2R2D- 2 axis is associated with an immunosuppressive tumor microenvironment, characterized by shifts in macrophage marker abundance.These findings reveal a previously uncharacterized function of HSPA8 as a modulator of alternative splicing, establishing the HSPA8- PPP2R2D- 2 axis as a critical driver of TNBC progression through dual mechanisms- direct apoptotic evasion and changes in the tumor immune microenvironment associated with shifts in macrophage markers.

## 2 Materials and methods

### 2.1 Cell lines and culture conditions

Human TNBC cell lines MDA-MB-231 (harboring TP53 R280K missense mutation) and HCC38 (TP53 wild-type) were obtained from Wuhan Procell Life Technology Co., Ltd. (Wuhan, China). HEK293T cells were obtained from the Cell Bank of the Chinese Academy of Sciences (Shanghai, China). All cell lines were authenticated by short tandem repeat (STR) profiling and tested negative for mycoplasma contamination. Cells were cultured in DMEM/F12 (1:1) complete medium supplemented with 10% fetal bovine serum (FBS; Gibco, USA) and 1% penicillin-streptomycin (Invitrogen, USA) at 37 °C in a humidified incubator with 5% CO_2_. Routine subculturing was performed at 2–3-day intervals using 0.25% EDTA-free trypsin, and all functional assays were conducted using cells in the logarithmic growth phase.

### 2.2 Transient transfection and stable cell line establishment

For transient transfection, HSPA8-targeting siRNA (si-HSPA8 3) and overexpression plasmids were synthesized by Guangzhou RiboBio Co., Ltd. (Guangzhou, China). Transfections were performed using Lipo 8000™ reagent (Beyotime, China) according to the manufacturer’s instructions. Briefly, cells seeded in 6-well plates were transfected with complexes formed by mixing 2.5 μg plasmid (or 50 nM siRNA) and 4 μL reagent in 125 μL Opti-MEM (Gibco, USA). Cells were collected for downstream applications 48 hours post-transfection.

For stable HSPA8 knockdown, cells were transduced with lentiviral vectors encoding short hairpin RNA (shRNA) targeting HSPA8 (shHSPA8) or non-targeting control (shCtrl). After 48 hours, cells were maintained under selection pressure using 2 μg/mL puromycin (InvivoGen, USA) for at least seven days. For functional rescue experiments, stable HSPA8-knockdown cells were subsequently infected with lentiviruses encoding Flag-tagged PPP2R2D-1 or PPP2R2D-2. Successful isoform expression was confirmed by anti-Flag western blot.

### 2.3 RNA extraction and quantitative real-time PCR

Total RNA was extracted from cultured cells using the FastPure^®^ Total RNA Isolation Kit V2 (Vazyme, China) according to the manufacturer’s protocol. One microgram of purified RNA was reverse-transcribed to cDNA using the HiScript III cDNA Synthesis Kit (Vazyme, China). Quantitative PCR was performed on a QuantStudio 5 instrument (Applied Biosystems, USA) using Taq Pro Universal SYBR qPCR Master Mix (Vazyme, China). The amplification protocol consisted of initial denaturation at 95 °C for 30 seconds, followed by 40 cycles of denaturation at 95 °C for 10 seconds and annealing/extension at 60 °C for 30 seconds. Each assay was performed with three technical replicates. GAPDH served as the reference gene for normalization, and relative gene expression levels were determined using the comparative 2^–ΔΔCt method. Primer sequences were: HSPA8 forward, 5’-ACCTACTCTTGTGTGGGTGTT-3’; HSPA8 reverse, 5’-GACATAGCTTGGAGTGGTTCG-3’; GAPDH forward, 5’-CCATCACCATCTTCCAGGAG-3’; GAPDH reverse, 5’-ATGATGACCCTTTTGGCTCC-3’.

### 2.4 Western blot analysis

Cells or tumor tissues were lysed in RIPA buffer (Beyotime, China) containing protease inhibitor cocktail (Roche, Switzerland). Protein concentrations were determined using the BCA Protein Assay Kit (Thermo Fisher Scientific, USA). Equal amounts of protein (20-30 μg) were separated by 10% SDS-PAGE and transferred to PVDF membranes (Millipore, USA). Membranes were blocked with 5% skim milk in TBST for 1 hour at room temperature, then incubated overnight at 4 °C with primary antibodies against HSPA8 (1:1000, Proteintech, China), FLAG (1:1000, Sigma-Aldrich, USA), BAX (1:1000, Cell Signaling Technology, USA), BCL-2 (1:1000, Cell Signaling Technology, USA), cleaved Caspase-3 (1:500, Cell Signaling Technology, USA), and GAPDH (1:5000, Proteintech, China). After washing with TBST, membranes were incubated with HRP-conjugated secondary antibodies (1:10000, Proteintech, China) for 1 hour at room temperature. Protein bands were visualized using an Ultra-Sensitive ECL substrate (Millipore, USA) and imaged with a ChemiDoc MP Imaging System (Bio-Rad, USA).

### 2.5 Cell proliferation assay

Cell proliferation was evaluated using the Enhanced Cell Counting Kit-8 (CCK-8; Beyotime, China). Cells were seeded into 96-well plates at a density of 2.5 × 10³ cells per well and cultured for 0, 24, 48, and 72 hours. At each time point, 10 μL of CCK-8 solution was added to each well, and plates were incubated for an additional 2 hours. Absorbance at 450 nm was measured using a microplate reader (Bio-Rad, USA).

### 2.6 Migration and invasion assays

Cell migration and invasion were assessed using Transwell chambers (24-well plates, 8.0 μm pore size; Corning, USA). For invasion assays, membranes were pre-coated with diluted Matrigel (BD Biosciences, USA). Cells (5×10^4^) suspended in serum-free medium were placed in the upper chamber, while the lower chamber contained complete medium with 10% FBS as a chemoattractant. After 24 hours incubation, non-migrated or non-invaded cells on the upper membrane surface were gently removed with a cotton swab. Cells that had traversed the membrane were fixed with 4% paraformaldehyde, stained with 0.1% crystal violet, and counted in five randomly selected fields under an inverted microscope (Olympus, Japan).

### 2.7 Apoptosis analysis by flow cytometry

Apoptosis was evaluated using the Annexin V-FITC/PI Apoptosis Detection Kit (BD Biosciences, USA) according to the manufacturer’s instructions. Cells (approximately 1×10^5^) were harvested, washed with cold PBS, and resuspended in binding buffer. After staining with Annexin V-FITC and PI for 15 minutes at room temperature in the dark, samples were analyzed on a BD Accuri C6 flow cytometer (BD Biosciences, USA). Data were processed using FlowJo software (version 10.0; Tree Star, USA). Early and late apoptotic cells were quantified and combined for statistical analysis.

### 2.8 RNA immunoprecipitation (RIP)

RIP assays were performed using the Magna RIP™ RNA-Binding Protein Immunoprecipitation Kit (Millipore, USA) according to the manufacturer’s protocol. Briefly, cell lysates prepared with RNase inhibitors were incubated overnight at 4 °C with 5 μg of HSPA8 antibody (Proteintech, China) or isotype control rabbit IgG (Millipore, USA). Protein A/G magnetic beads were then added and incubated for 1 hour at 4 °C. After stringent washes, immunoprecipitated materials were digested with Proteinase K. RNA specifically bound to HSPA8 was isolated and subjected to RT-qPCR analysis to detect PPP2R2D pre-mRNA enrichment.

### 2.9 Splicing isoform analysis by semi-quantitative RT-PCR

To assess the relative levels of PPP2R2D splice variants, semi-quantitative RT-PCR was performed using primers flanking exon 2. The PCR conditions were as follows: initial denaturation at 95 °C for 3 min; 28 cycles of 95 °C for 30 s, 58 °C for 30 s, and 72 °C for 90 s; and a final extension at 72 °C for 5 min. GAPDH (25 cycles) was used as an internal control. A 22–32 cycle gradient pre-experiment was performed to ensure that the 28-cycle amplification was within the linear range and had not reached the plateau phase. PCR products were resolved on 2% agarose gels and stained with GeneRed nucleic acid dye (Biotium, USA). Gel images were captured using a Gel Doc XR+ System (Bio-Rad, USA). Band intensities corresponding to PPP2R2D-2 (full-length) and PPP2R2D-1 (exon 2-skipped) were quantified using ImageJ software (NIH, USA), and the splicing ratio was calculated accordingly. The PSI was calculated as PSI = PPP2R2D-2/(PPP2R2D-1 + PPP2R2D-2) × 100%. All experiments were independently repeated three times (n=3).

### 2.10 RNA sequencing and bioinformatic analysis

Total RNA was extracted from HCC38 cells with HSPA8 knockdown (shHSPA8) and control (shCtrl) in three independent biological replicates (n=3) using TRIzol reagent(Invitrogen, USA). Library preparation and sequencing were performed by Novogene (Beijing, China) on an Illumina NovaSeq 6000 platform with 150 bp paired-end reads. Sequencing reads were aligned to the GRCh38 human reference genome using HISAT2. Differential alternative splicing analysis was performed with rMATS (v4.1.0), with significant events defined by |ΔPSI| > 10% and FDR < 0.05. Splicing patterns and read coverage for the PPP2R2D locus were visualized using the Integrative Genomics Viewer (IGV). Differentially expressed genes were determined from normalized read counts with thresholds of |log_2_FC| > 1 and P < 0.05.

### 2.11 Public database analysis

GEPIA (http://gepia.cancer-pku.cn) was used for KEGG pathway enrichment analysis of HSPA8-correlated genes. TSVdb (http://tsvdb.com) was employed to analyze PPP2R2D isoform expression patterns during breast cancer progression. Kaplan-Meier survival analysis for PPP2R2D isoforms was performed using public microarray datasets. TIMER (https://cistrome.shinyapps.io/timer) and CIBERSORT algorithms were applied to TCGA TNBC data to evaluate correlations between HSPA8 expression and immune cell infiltration.

### 2.12 *In vivo* tumor xenograft studies

Four-week-old female BALB/c nude mice were obtained from the Laboratory Animal Center of Xinjiang Medical University and maintained under specific pathogen-free conditions. All animal experiments were approved by the Animal Ethics Committee of the Affiliated Cancer Hospital of Xinjiang Medical University (Approval No. XJZLL2024-0123). Stable MDA-MB-231 cell lines (shCtrl, shHSPA8, shHSPA8 + PPP2R2D-1, shHSPA8 + PPP2R2D-2) were harvested and resuspended at a density of 5×10^6^ cells in 100 μL of a 1:1 mixture of PBS and Matrigel (BD Biosciences, USA). The cell suspension was orthotopically injected into the right mammary fat pad of each mouse (5 mice per group). Tumor dimensions were measured every 3–4 days using calipers, and tumor volume was calculated according to the formula: volume = (length × width²) × 0.5. After 5 weeks, mice were euthanized, and tumors were excised, weighed, and processed for further analysis.

### 2.13 Histopathological and immunohistochemical analysis

Mouse tumor tissues were fixed in 4% paraformaldehyde, embedded in paraffin, and sectioned at 5 μm thickness. For H&E staining, sections were deparaffinized, rehydrated, stained with hematoxylin for 5 minutes and eosin for 1 minute, then dehydrated and mounted with neutral balsam. For immunohistochemical analysis of macrophage markers, sections were incubated with anti-CD86 (1:200, Abcam, UK) or anti-CD206 (1:200, Abcam, UK) antibodies overnight at 4 °C, followed by HRP-conjugated secondary antibodies. Staining was visualized using a DAB kit (Vector Laboratories, USA), and images were captured under an optical microscope (Olympus, Japan). Positively stained cells were counted in five randomly selected fields per section.

### 2.14 Statistical analysis

All *in vitro* assays were performed with at least three biological replicates. Results are presented as mean ± standard deviation (SD). Statistical analyses were conducted using GraphPad Prism software (version 8.0.2; GraphPad, USA). Differences between two groups were assessed using two-tailed unpaired Student’s t-test. For comparisons among three or more groups, one-way analysis of variance (ANOVA) was applied, followed by Tukey’s *post hoc* test for multiple comparisons. Statistical significance was set at P < 0.05 (P < 0.05, P < 0.01, P < 0.001, P < 0.0001).

## 3 Results

### 3.1 HSPA8 is upregulated in TNBC and promotes malignant phenotypes

We first examined HSPA8 protein expression across a panel of breast cancer cell lines by western blot. HSPA8 expression was markedly elevated in TNBC cell lines, with the highest levels observed in HCC38 and MDA-MB-231 cells (**Figure 1A**). Based on this, we selected these two TNBC cell lines for subsequent gain- and loss-of-function studies. qPCR and western blot confirmed successful HSPA8 overexpression and knockdown at both mRNA and protein levels (**Figure 1B**). Three siRNAs were tested, and si-HSPA8–3 was selected for all subsequent experiments due to its superior knockdown efficacy.

**Figure 1:**
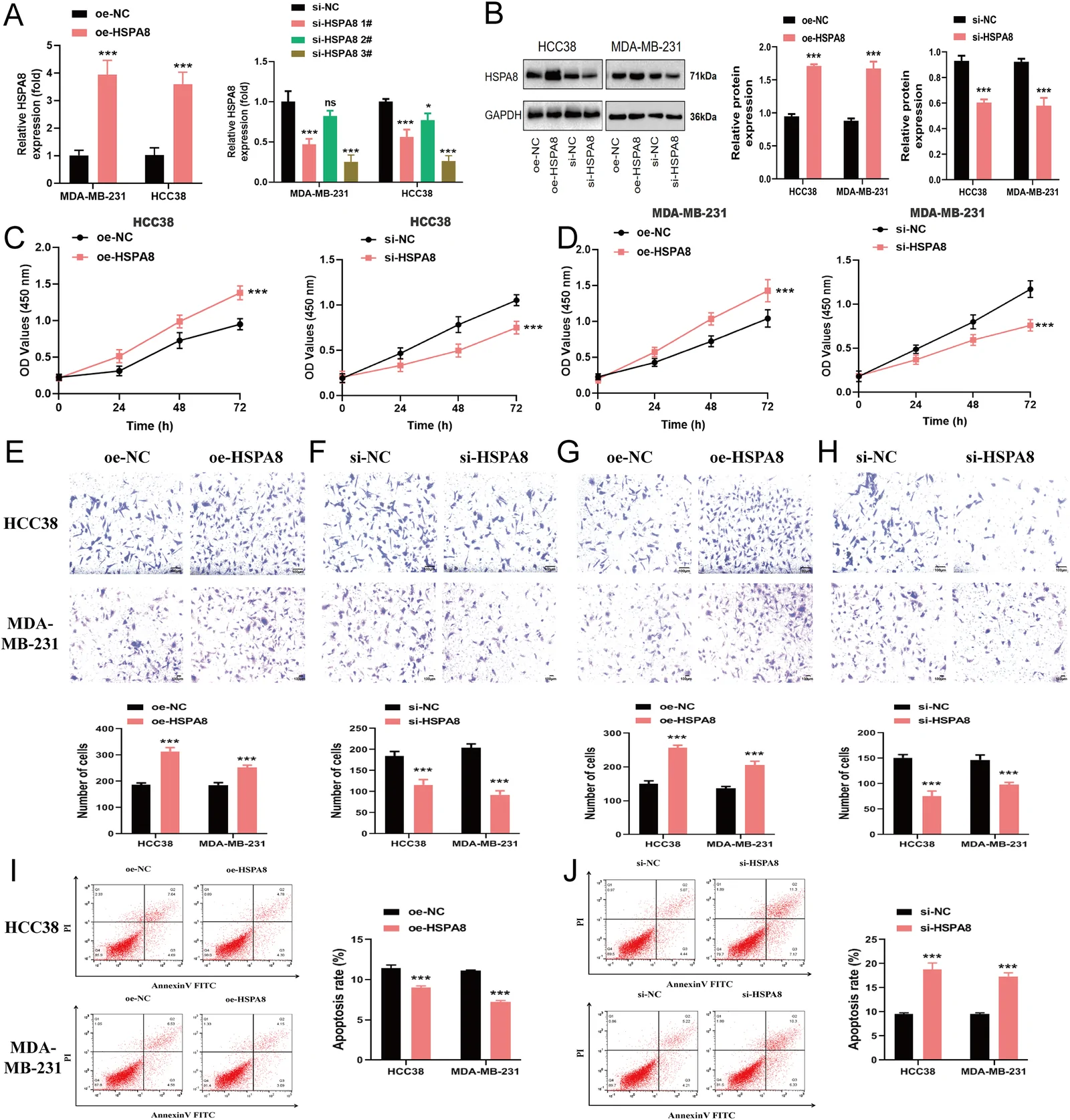
HSPA8 promotes malignant phenotypes and suppresses apoptosis in TNBC cells. **(A)** RT-qPCR analysis of HSPA8 mRNA expression levels in MDA-MB-231 and HCC38 cells following transfection with overexpression plasmids (oe-HSPA8) or siRNAs against HSPA8 (si-HSPA8 1, 2, 3), confirming the efficiency of overexpression and knockdown. ***P < 0.001. **(B)** Western blot analysis of HSPA8 protein expression in HCC38 and MDA-MB-231 cells. The bands and bar graphs respectively show the baseline protein levels and the confirmation of overexpression (oe-HSPA8) and knockdown (si-HSPA8) efficiency at the protein level. GAPDH was used as an internal control. ***P < 0.001. **(C)** CCK-8 assays showing the effects of HSPA8 overexpression or knockdown on the proliferation of HCC38 cells (labeled in the figure). **(D)** CCK-8 assays showing the effects of HSPA8 overexpression or knockdown on the proliferation of MDA-MB-231 cells (labeled in the figure). ***P < 0.001. **(E, F)** Transwell migration assays demonstrating the effects of HSPA8 modulation on migratory capacity. ***P < 0.001. Scale bar, 100 μm. **(G, H)** Transwell invasion assays showing the effects of HSPA8 modulation on invasive ability. ***P < 0.001. Scale bar, 100 μm. **(I, J)** Flow cytometry analysis of apoptosis upon HSPA8 overexpression or knockdown. *P < 0.05, ***P < 0.001; ns, not significant.

Functional assays demonstrated that HSPA8 overexpression significantly enhanced the proliferative capacity of TNBC cells, while HSPA8 knockdown suppressed proliferation (**Figures 1C, D**). Transwell assays revealed that HSPA8 overexpression promoted both migration and invasion, whereas HSPA8 knockdown markedly inhibited these aggressive phenotypes (**Figures 1E–H**). Flow cytometry analysis showed that HSPA8 overexpression reduced apoptosis, and conversely, HSPA8 knockdown significantly increased apoptotic rates (**Figures 1I, J**). These results establish HSPA8 as an oncogenic driver in TNBC, promoting proliferation, migration, invasion, and apoptotic resistance.

### 3.2 HSPA8 modulates alternative splicing of PPP2R2D

To elucidate the molecular mechanisms underlying HSPA8-mediated TNBC progression, we performed KEGG pathway enrichment analysis of HSPA8-correlated genes using the GEPIA database. Notably, splicing-related pathways were among the enriched categories (**Figure 2A**). Although this analysis was based on differentially expressed genes rather than direct RNA targets, it prompted us to investigate whether HSPA8 might influence splicing regulation. We therefore conducted RNA-seq on HCC38 cells with HSPA8 knockdown (shHSPA8) versus control (shCtrl) in three independent biological replicates.Differential expression analysis identified 152 DEGs (52 upregulated, 100 downregulated) upon HSPA8 silencing (**Figures 2B, C**). Global analysis of alternative splicing events revealed that HSPA8 depletion induced a distinct set of alternative splicing changes without altering the total number of events, indicating that HSPA8 acts as a splicing modulator that reprograms specific splicing patterns rather than a global disruptor of the splicing machinery (**Figure 2D**).

**Figure 2:**
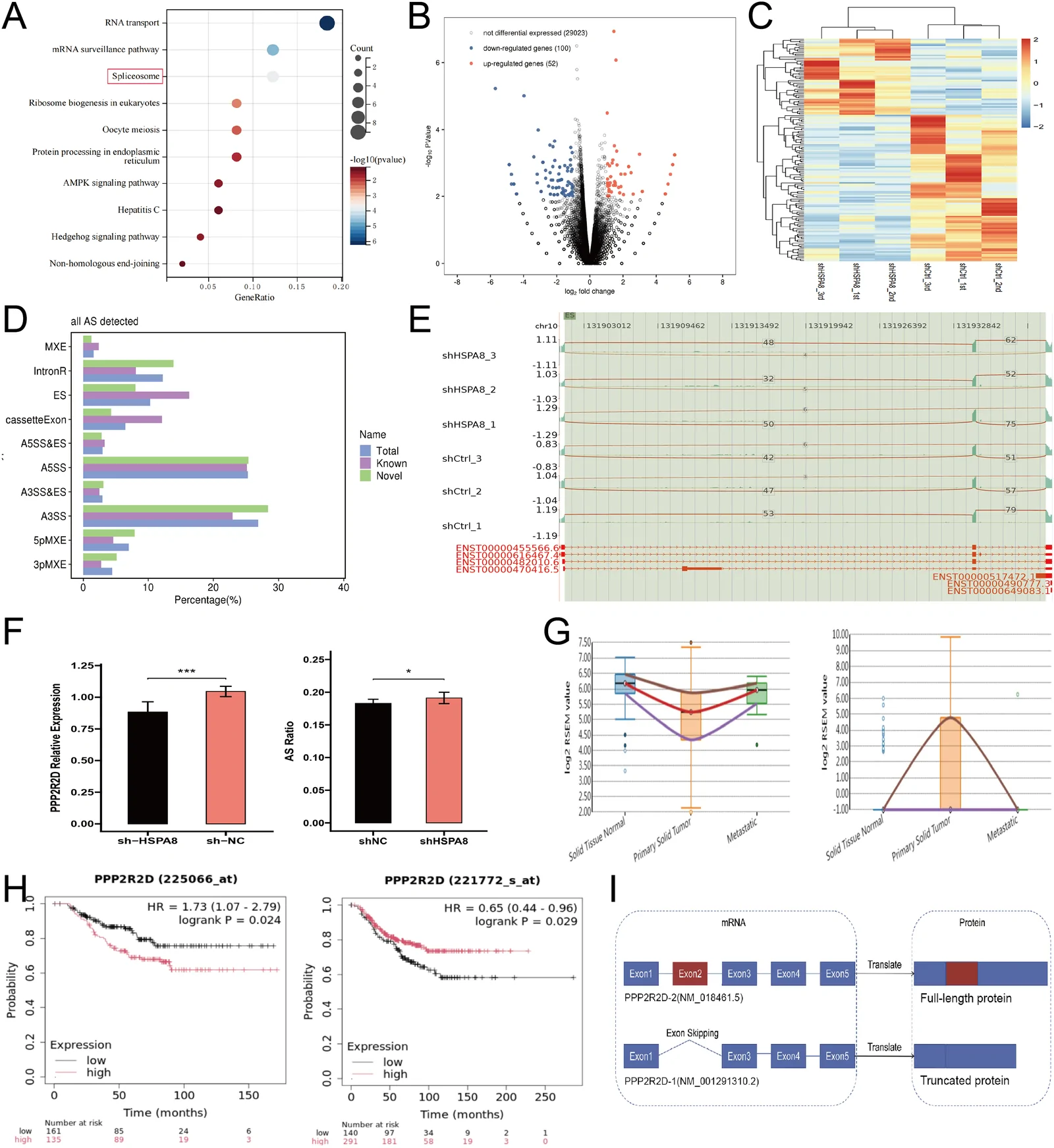
HSPA8 regulates alternative splicing of PPP2R2D, generating functionally divergent isoforms. **(A)** KEGG pathway enrichment analysis of HSPA8-correlated genes in the GEPIA database reveals enrichment of splicing-related pathways among the differentially expressed genes, prompting further investigation into potential splicing regulatory functions. **(B)** Volcano plot displaying DEGs following HSPA8 knockdown (|log_2_FC| > 1, P < 0.05). **(C)** Hierarchical clustering of DEGs in shHSPA8 vs. shCtrl samples. **(D)** Distribution and quantitative comparison of alternative splicing event types between shHSPA8 and shCtrl samples. The total number of events remained comparable between groups, suggesting selective reprogramming rather than global splicing disruption. **(E)** IGV snapshot of PPP2R2D locus showing altered read coverage upon HSPA8 knockdown. Red box and arrow highlight the specific exon (Exon 2) involved in the alternative splicing event to improve interpretability. **(F)** RT-qPCR validation of total PPP2R2D mRNA levels and exon 2 skipping ratio. Data points represent independent biological replicates (n=3), and values are shown as mean ± SD. Statistical significance was determined using an unpaired two-tailed Student’s t-test. ***P < 0.001, *P < 0.05. **(G)** Schematic diagram of PPP2R2D isoforms: full-length PPP2R2D-2 and truncated PPP2R2D-1 (exon 2 skipping). **(H)** TSVdb analysis showing opposite expression trends of PPP2R2D isoforms during breast cancer progression. **(I)** Schematic diagram illustrating alternative splicing events of the PPP2R2D gene. Exon skipping of Exon 2 generates two distinct mRNA isoforms: full-length PPP2R2D-2 (NM_018461.5) encoding full-length protein, and truncated PPP2R2D-1 (NM_001291310.2) producing truncated protein, which exhibit opposing prognostic effects as shown in Kaplan-Meier survival analysis. These opposing clinical outcomes may reflect the differential functional roles of the two isoforms.The data are presented as the means ± SD of three independent experiments (RNA-seq, n=3).

Among the differentially spliced genes, PPP2R2D—a regulatory subunit of PP2A—emerged as a significant hit. Integrative Genomics Viewer (IGV) visualization showed marked alterations in read coverage at the PPP2R2D locus upon HSPA8 knockdown, particularly affecting exon 2 (**Figure 2E**). RT-qPCR validation confirmed that HSPA8 knockdown significantly reduced total PPP2R2D mRNA levels while increasing the exon 2 skipping ratio (PPP2R2D-1/PPP2R2D-2) (**Figure 2F**). Database mining revealed that PPP2R2D generates two major isoforms through alternative splicing: the full-length PPP2R2D-2 (NM_018461.5, including exon 2) and the truncated PPP2R2D-1 (NM_001291310.2, resulting from exon 2 skipping) (**Figure 2G**). Analysis of TSVdb database demonstrated that these two isoforms exhibit opposite expression trends during breast cancer progression: PPP2R2D-1 expression gradually increased, while PPP2R2D-2 expression gradually decreased as tumor stage advanced (**Figure 2H**). Strikingly, Kaplan-Meier survival analysis based on public microarray data revealed that high expression of the two isoforms correlates with opposing prognostic outcomes in breast cancer patients (**Figure 2I**). These findings suggest that PPP2R2D isoforms may possess functionally distinct roles in TNBC, with only PPP2R2D-2 retaining pro-survival activity.

### 3.3 HSPA8 is physically associated with PPP2R2D pre-mRNA and promotes PPP2R2D-2 expression

To determine whether HSPA8 regulates PPP2R2D splicing through RNA association, we performed RNA immunoprecipitation (RIP) assays. HSPA8 antibody significantly enriched PPP2R2D pre-mRNA compared to control IgG, demonstrating that HSPA8 is physically associated with PPP2R2D pre-mRNA within a ribonucleoprotein complex *in vivo* (**Figure 3A**). While this establishes a physical association, it does not distinguish between direct binding or indirect binding through other bridging proteins, which remains to be investigated.

**Figure 3:**
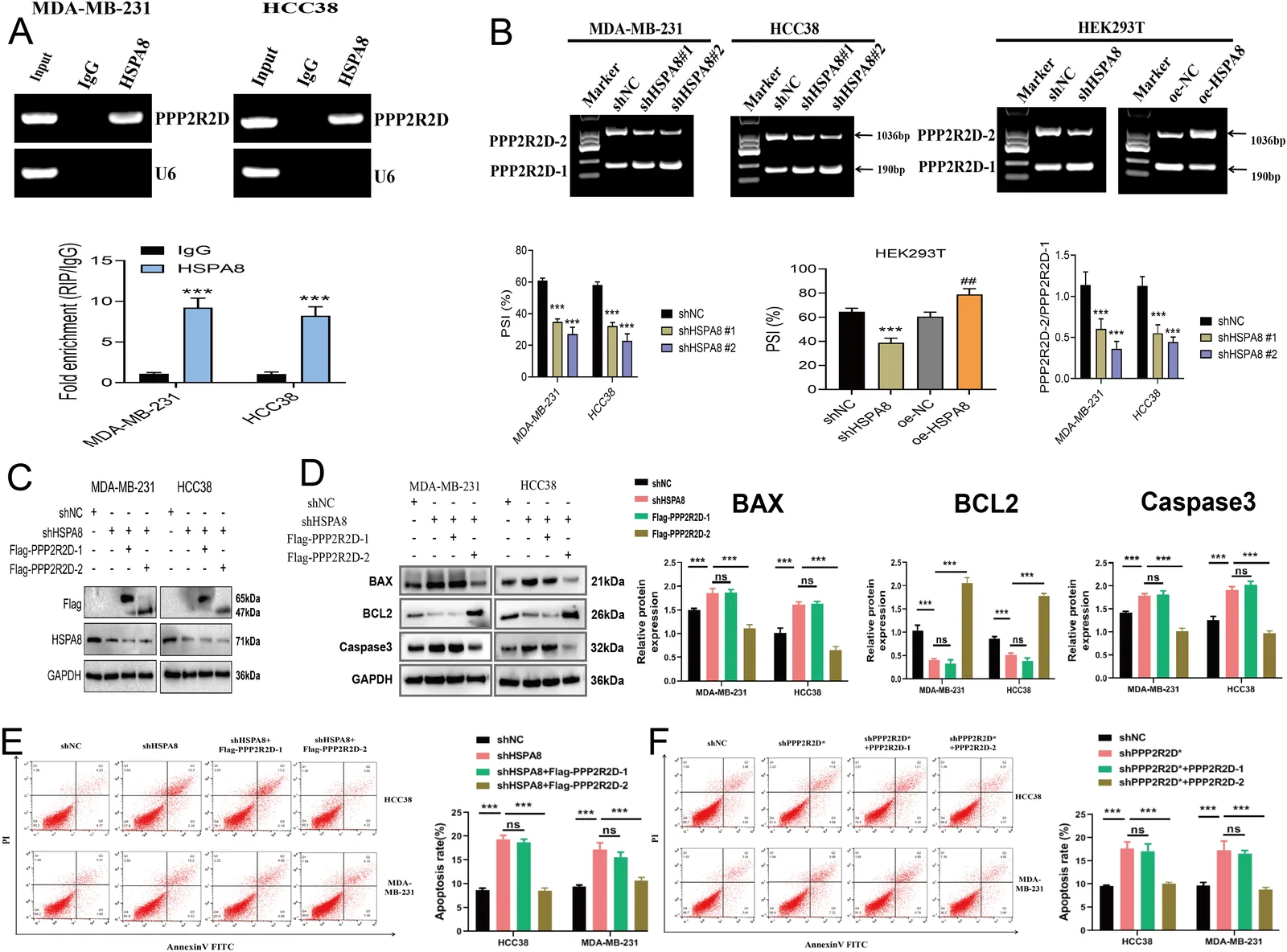
HSPA8 is physically associated with PPP2R2D pre-mRNA within a complex, and PPP2R2D-2 is the functional isoform mediating its anti-apoptotic effect. **(A)** RIP assay showing that HSPA8 is physically associated with PPP2R2D pre-mRNA within a complex *in vivo* in MDA-MB-231 and HCC38 cells (cell lines are labeled in the figure). IgG serves as negative control. The bar graph shows the fold enrichment of PPP2R2D mRNA relative to IgG, and the data are presented as the means ± SD of three independent biological replicates. ***P < 0.001. **(B)** Semi-quantitative RT-PCR analysis of PPP2R2D splicing in MDA-MB-231, HCC38, and HEK293T cells using primers spanning Exon 2 (1036 bp for PPP2R2D-2 and 190 bp for PPP2R2D-1).The PCR was performed for 28 cycles, which was validated to be within the linear amplification range by a 22–32 cycle gradient pre-experiment. Band intensities were quantified using ImageJ, and the percent spliced in (PSI) values and PPP2R2D-2/PPP2R2D-1 ratios are shown in the bar graphs, which were derived from three independent biological replicates, and were quantitatively analyzed in both TNBC models (MDA-MB-231 and HCC38). The splicing shift was additionally validated via RT-qPCR. ***P < 0.001, *P < 0.05; ns, not significant. **(C)** Western blot confirming HSPA8 knockdown and isoform-specific rescue (Flag-PPP2R2D-1 and Flag-PPP2R2D-2) in MDA-MB-231 and HCC38 cells. GAPDH serves as an internal control. **(D)** Western blot analysis of apoptosis-related proteins. HSPA8 knockdown increases BAX and cleaved Caspase-3, decreases BCL-2; these effects are specifically reversed by PPP2R2D-2 re-expression. The bar graphs show the quantification of three independent experiments. ***P < 0.001; ns, not significant. **(E)** Flow cytometry analysis of apoptosis in HCC38 and MDA-MB-231 cells. Increased apoptosis upon HSPA8 knockdown is rescued only by PPP2R2D-2. ***P < 0.001; ns, not significant. **(F)** Following pan-PPP2R2D knockdown, only PPP2R2D-2 reverses the pro-apoptotic phenotype in HCC38 and MDA-MB-231 cells. ***P < 0.001; ns, not significant.

To quantitatively validate the HSPA8-dependent PPP2R2D splicing event in both TNBC models, we performed RT-qPCR and semi-quantitative RT-PCR. The results confirmed that HSPA8 knockdown shifted the splicing pattern toward the truncated PPP2R2D-1 isoform in both MDA-MB-231 and HCC38 cells (**Figure 3B**). The percent spliced in (PSI) values and the PPP2R2D-2/PPP2R2D-1 ratios were quantitatively analyzed using ImageJ in both TNBC models based on three independent biological replicates, validating the RNA-seq findings. Western blot confirmed successful HSPA8 knockdown and isoform-specific rescue in stable cell lines, confirming that PPP2R2D functions downstream of HSPA8 (**Figure 3C**).

### 3.4 PPP2R2D-2 mediates the anti-apoptotic function of HSPA8

We next performed functional rescue experiments to determine which PPP2R2D isoform mediates HSPA8’s biological effects. Western blot analysis showed that HSPA8 knockdown significantly increased pro-apoptotic BAX and cleaved Caspase-3 levels while decreasing anti-apoptotic BCL-2 expression. Notably, these alterations were specifically reversed by re-expression of PPP2R2D-2, whereas PPP2R2D-1 had no significant effect (**Figure 3D**). Flow cytometry analysis confirmed that the increased apoptosis induced by HSPA8 knockdown was rescued only by PPP2R2D-2, not by PPP2R2D-1 (**Figure 3E**). To further validate isoform-specific functions, we knocked down all PPP2R2D isoforms and performed rescue experiments with each isoform individually. Reexpression of PPP2R2D-2, but not PPP2R2D-1, effectively reversed the pro-apoptotic phenotype caused by pan-PPP2R2D knockdown (**Figure 3F**). These data indicate that PPP2R2D-1 lacks the pro-survival function of PPP2R2D-2, rather than actively exerting an opposing effect. Therefore, PPP2R2D-2 is established as the key functional isoform mediating HSPA8’s anti-apoptotic effects through modulation of the mitochondrial apoptosis pathway.

### 3.5 HSPA8-PPP2R2D-2 axis promotes TNBC tumorigenesis *in vivo*

To validate our findings *in vivo*, we established subcutaneous xenograft models using MDA-MB-231 cells with stable HSPA8 knockdown and isoform-specific rescue. HSPA8 silencing significantly suppressed tumor growth compared to control group. Remarkably, re-expression of PPP2R2D-2 effectively rescued this suppressive effect, restoring tumor growth to near-control levels, whereas PPP2R2D-1 had no significant effect (**Figure 4A**). Final tumor weights confirmed this pattern (**Figure 4A**). Western blot analysis of xenograft tumor tissues confirmed successful manipulation of HSPA8 and PPP2R2D isoform expression (**Figure 4B**). Histopathological examination by H&E staining revealed that HSPA8 knockdown led to less aggressive tumor morphology characterized by more regular, orderly cells, which was reverted to a malignant phenotype with nuclear pleomorphism and disordered arrangement only in the PPP2R2D-2 rescue group (**Figure 4C**). These *in vivo* data solidify the conclusion that HSPA8 promotes TNBC tumorigenesis predominantly through sustaining PPP2R2D-2 isoform expression.

**Figure 4:**
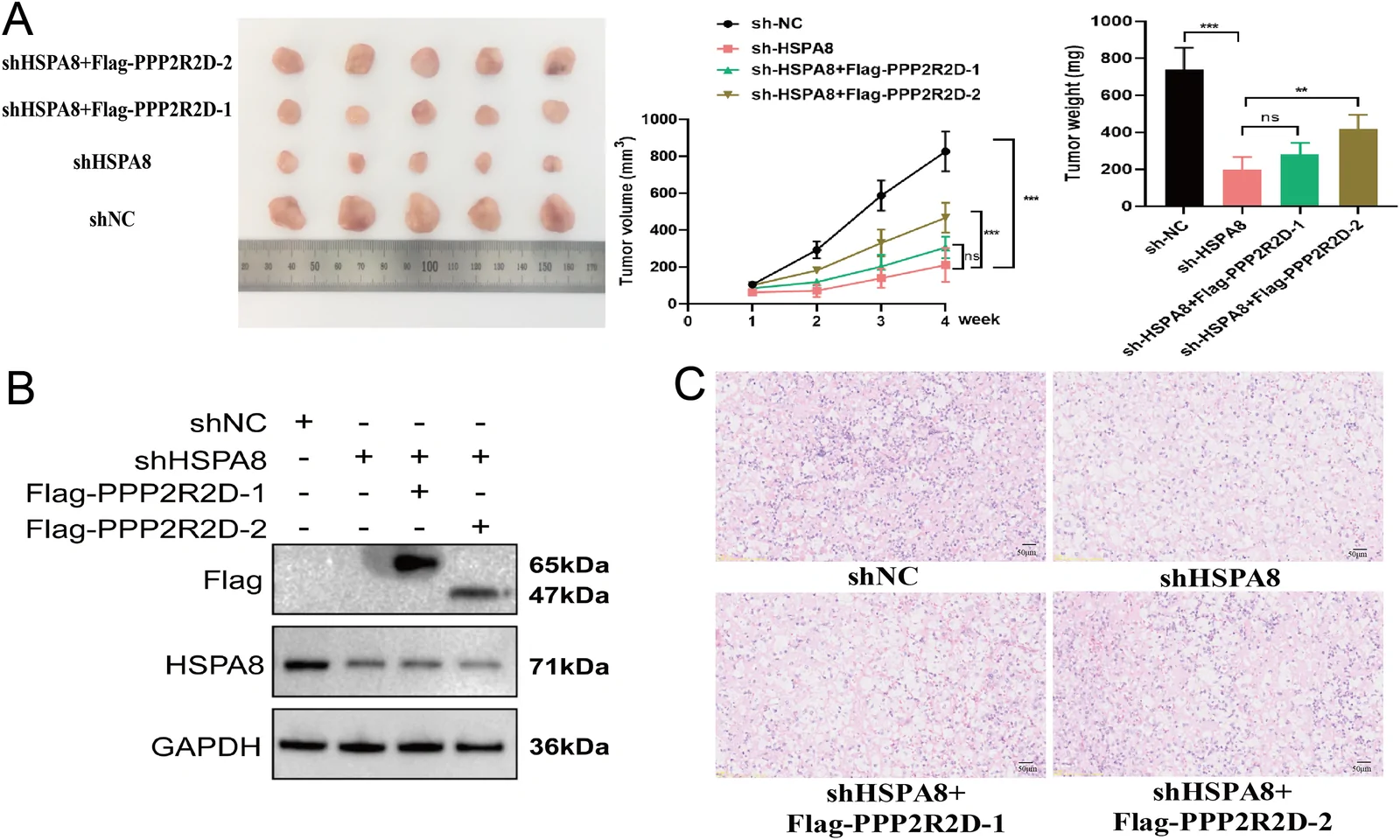
The HSPA8-PPP2R2D-2 axis promotes TNBC tumorigenesis *in vivo*. **(A)** Tumor growth curves and final tumor weights in a subcutaneous xenograft model (n=5 mice per group). HSPA8 knockdown significantly suppresses tumor growth; however, re-expression of PPP2R2D-2, but not PPP2R2D-1, specifically rescues this effect. ***P < 0.001, **P < 0.01; ns, not significant. **(B)** Western blot analysis of xenograft tumor tissues confirming the knockdown of HSPA8 and the specific re-expression of Flag-PPP2R2D-1 or Flag-PPP2R2D-2. GAPDH was used as an internal control. **(C)** Representative H&E staining of tumor sections from each group. PPP2R2D-2 rescue restores the malignant morphological features. Scale bar, 50 μm. The data are presented as the means ± SD of three independent experiments (n=5 mice per group).

### 3.6 HSPA8- PPP2R2D- 2 axis is associated with changes in macrophage polarization markers in the tumor immune microenvironment

Given the established role of tumor microenvironment in cancer progression, we investigated whether HSPA8 influences immune cell infiltration. TIMER algorithm analysis of TCGA TNBC data revealed that high HSPA8 expression significantly correlated with increased macrophage infiltration (**Figure 5A**). CIBERSORT analysis further demonstrated that HSPA8 expression was specifically associated with M2 (pro-tumor) macrophage polarization, but not M1 (anti-tumor) macrophages (**Figure 5B**). To validate these findings, we performed immunohistochemical staining of M1 (CD86^+^) and M2 (CD206^+^) macrophages in xenograft tumor tissues. HSPA8 knockdown significantly increased anti-tumor M1 macrophages and decreased pro-tumor M2 macrophages. Remarkably, re-expression of PPP2R2D-2 was associated with the restoration of the M2-marker dominant state, whereas PPP2R2D-1 had no effect (**Figure 5C**). These findings suggest that the HSPA8- PPP2R2D- 2 axis is associated with changes in the tumor immune microenvironment, characterized by significant changes in macrophage marker abundance. However, these correlative data do not directly establish active macrophage polarization.

**Figure 5:**
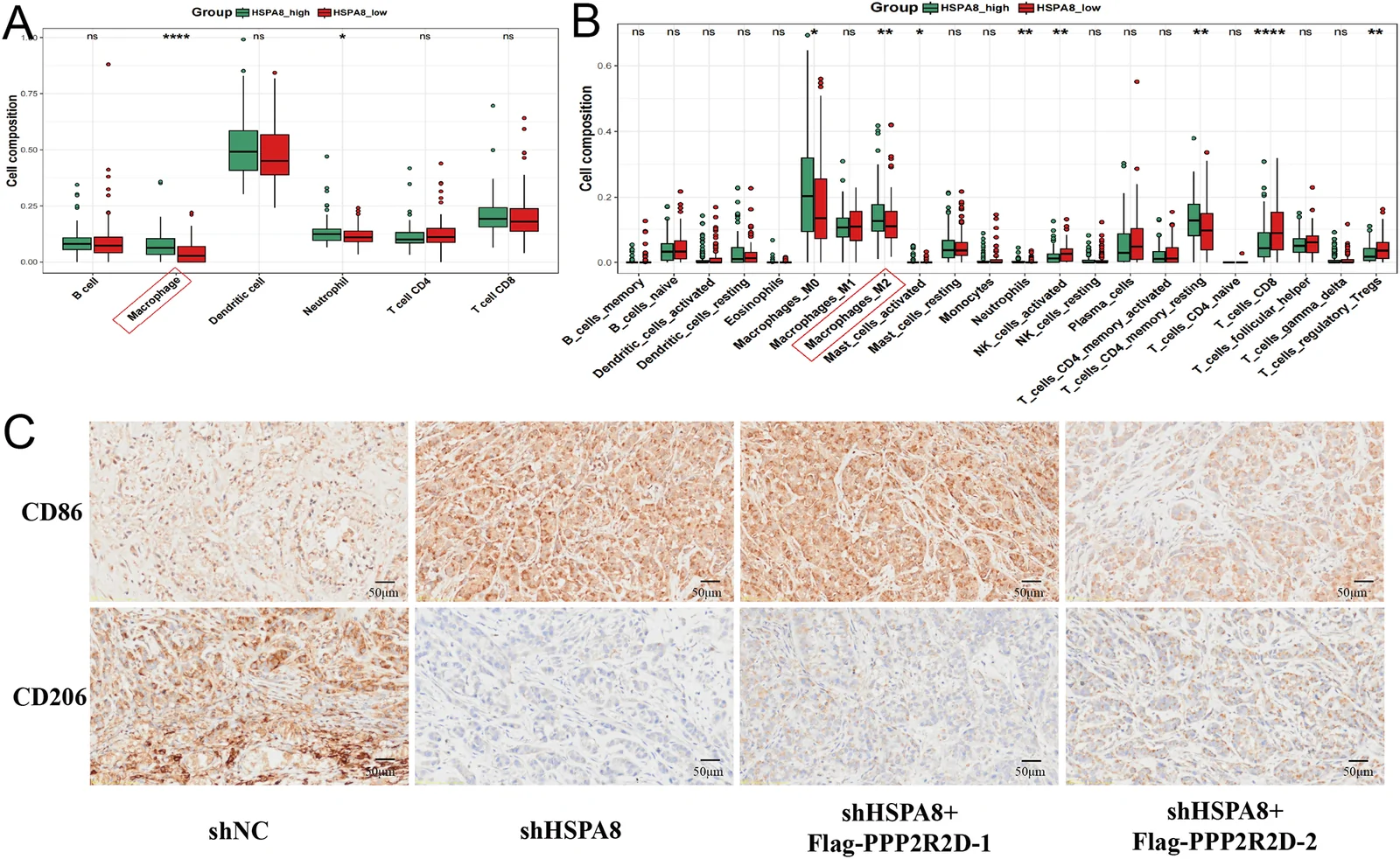
The HSPA8- PPP2R2D- 2 axis is associated with shifts in macrophage markers in the tumor immune microenvironment. **(A)** TIMER analysis of TCGA TNBC data showing the correlation between HSPA8 expression levels and immune cell infiltration. Red box highlights the significantly altered immune cell population (Macrophage). ****P < 0.0001; ns, not significant. **(B)** CIBERSORT analysis showing the specific association of HSPA8 expression with macrophage subtypes. Red boxes highlight the significant changes in M0 and M2 macrophage polarization. *P < 0.05, **P < 0.01; ns, not significant. **(C)** Representative immunohistochemical (IHC) staining of M1 (CD86+) and M2 (CD206+) macrophages HSPA8 knockdown significantly increases M1 markers and decreases M2 markers, whereas re-expression of PPP2R2D-2, but not PPP2R2D-1, restores the M2 marker-dominant state. These data reflect changes in macrophage marker abundance.

## 4 Discussion

In this study, we uncover a paradigm-shifting function for HSPA8 in TNBC. We demonstrate that HSPA8, traditionally viewed as a molecular chaperone, functions as a modulator of alternative splicing of PPP2R2D. This regulation favors the production of the full-length PPP2R2D-2 isoform, which in turn drives TNBC progression through two synergistic mechanisms: direct suppression of mitochondrial apoptosis and changes in the tumor immune microenvironment associated with shifts in macrophage markers. Our findings not only establish the HSPA8-PPP2R2D-2 axis as a critical driver of TNBC aggressiveness but also reveal a novel layer of complexity in cancer biology where a single splicing event coordinates both cell-intrinsic survival and cell-extrinsic immune microenvironment alterations.

The identification of HSPA8 as a modulator of cancer-related splicing represents a significant conceptual advance. While classical chaperones have been primarily studied for their roles in maintaining proteostasis, recent evidence hints at their involvement in RNA metabolism (17). Our RIP assays demonstrate that HSPA8 is incorporated into an RNA-protein complex containing PPP2R2D pre-mRNA *in vivo*, and the selective splicing changes observed upon HSPA8 knockdown (**Figure 2D**) argue against a global, nonspecific effect on the splicing machinery. However, we acknowledge that HSPA8 lacks canonical RNA-binding domains such as RRM or KH domains, and thus the precise nature of this interaction. Our current data support an association within the complex, rather than direct binding; therefore, whether HSPA8 directly binds RNA in a sequence- or structure- specific manner, or acts as part of a larger splicing regulatory complex through bridging factors such as hnRNPs or SR proteins requires further investigation. Nonetheless, the specificity of the splicing outcome (affecting PPP2R2D exon 2 retention without globally disrupting splicing) suggests that HSPA8 functions as a selective splicing modulator, likely through direct or indirect interaction with the splicing machinery, positioning HSPA8 within the growing family of splicing regulators whose dysregulation contributes to the aberrant splicing landscape characteristic of cancer (6). Future studies employing RNA pull-down or electrophoretic mobility shift assays with recombinant HSPA8 protein will be necessary to resolve the direct versus indirect nature of this interaction and to elucidate the molecular determinants of HSPA8-RNA recognition, which could pave the way for developing small-molecule inhibitors that specifically disrupt its splicing regulatory function without affecting its essential chaperone activity.

Beyond its canonical chaperone functions, HSPA8 is increasingly recognized for non-proteostatic roles. As a member of the HSP70 family, it participates in RNA metabolism and has been implicated in viral entry through interactions with both protein and RNA components (11). The broader concept that molecular chaperones can moonlight as RNA-binding proteins is now well established (17), providing a conceptual framework for our finding that HSPA8 modulates PPP2R2D alternative splicing.

The discovery that PPP2R2D generates two functionally distinct isoforms through HSPA8-mediated splicing adds a new dimension to our understanding of PP2A biology. The full-length PPP2R2D-2 isoform promotes apoptotic resistance and tumor progression, whereas the truncated PPP2R2D-1 lacks these functions, rather than actively opposing them. This phenomenon—where a single gene produces functionally distinct isoforms through alternative splicing—has been documented for other cancer-associated genes including BCL2L1, KLF6, and MBNL1 (18, 19). The opposing expression trends of PPP2R2D isoforms during tumor progression and their correlation with contrasting patient survival outcomes underscore the clinical significance of this splicing switch. These opposing clinical outcomes may reflect the differential functional roles of the two isoforms. Mechanistically, we demonstrate that PPP2R2D-2 modulates the mitochondrial apoptosis pathway by regulating the BAX/BCL-2 ratio, thereby controlling the activation of executioner caspase-3. Although p53 is a critical tumor suppressor frequently dysregulated in cancer (20) and HSPA8 has been implicated in p53 folding and stabilization (21), MDA-MB-231 cells harbor mutant p53 (R280K) that lacks transcriptional activity. Therefore, the observed changes in BCL-2 expression upon PPP2R2D-2 manipulation strongly suggest that this regulation occurs through p53-independent mechanisms, distinguishing the present findings from the classical chaperone function of HSPA8. The precise molecular intermediates linking PPP2R2D-2 to BCL-2 transcriptional or post-translational regulation—potentially through PP2A-mediated dephosphorylation of key transcription factors or signaling kinases—represent an important area for future investigation.

Perhaps the most clinically relevant finding of our study is that the HSPA8-PPP2R2D-2 axis extends beyond tumor cell-autonomous effects to modulate the tumor immune microenvironment. We show that HSPA8 knockdown is associated with a shift in macrophage markers toward an anti-tumor M1 phenotype, while PPP2R2D-2 re-expression is associated with the restoration of an M2-marker dominant state. Tumor-associated macrophages (TAMs), particularly M2-polarized macrophages, are well-known promoters of tumor progression, angiogenesis, immunosuppression, and therapy resistance (22). The ability of cancer cells to actively remodel their microenvironment is increasingly recognized as a hallmark of malignancy (23). Our findings suggest a potential crosstalk between intrinsic splicing alterations in tumor cells and the immune landscape, establishing a feed-forward loop that sustains tumor progression. While the molecular mediators of this tumor-immune crosstalk require further elucidation, our data suggest that the HSPA8-PPP2R2D-2 axis may represent a potential target for future intervention, although such therapeutic implications, particularly regarding immune checkpoint blockade, require further validation in immunocompetent models. It is important to note that HSPA8 can also be secreted or localized to the cell membrane and extracellular vesicles (12), where it may exert immunomodulatory functions distinct from its intracellular roles (13). Our study focuses on the intracellular, nuclear function of HSPA8 in splicing regulation. The relationship between secreted HSPA8 and the intracellular splicing machinery remains to be defined, and future studies should investigate whether secreted HSPA8 can be internalized by recipient cells and influence splicing in a paracrine manner.

Our findings have several translational implications. First, the identification of PPP2R2D-2 as a critical pro-survival isoform suggests that strategies to shift splicing toward PPP2R2D-1 (e.g., using antisense oligonucleotides) might have therapeutic benefit. Second, targeting the RNA-binding function of HSPA8 with small molecules could disrupt the entire oncogenic axis. A critical challenge for therapeutic targeting is the selectivity of splicing modulation. Since HSPA8 is a constitutively expressed chaperone essential for general proteostasis, global inhibition would likely cause substantial toxicity. Several strategies could achieve specificity for the PPP2R2D splicing switch. First, antisense oligonucleotides (ASOs) designed to target the PPP2R2D exon 2 splice sites could directly modulate splicing without inhibiting HSPA8 itself, an approach that has shown promise in other splicing-driven cancers (19). Second, small-molecule splicing modulators that selectively disrupt the interaction between HSPA8 and the splicing machinery—rather than its chaperone activity—could provide a more targeted intervention (5). These approaches warrant future investigation to translate our findings toward clinical application. Third, given the association between HSPA8 expression and M2 macrophage markers, combining HSPA8-targeted therapies with immune checkpoint blockade may yield synergistic effects, although this hypothesis requires further validation in immunocompetent models. The recent success of immunotherapy in a subset of TNBC patients (24) underscores the importance of understanding mechanisms that shape the immune landscape.

Several limitations of this study should be acknowledged. First, the precise molecular determinants of HSPA8-RNA recognition remain to be elucidated. Second, while we have identified PPP2R2D as a key downstream target, HSPA8 likely regulates additional splicing events that contribute to TNBC progression. Genome-wide approaches such as eCLIP-seq will be necessary to comprehensively map the HSPA8-RNA interactome. Third, our *in vivo* studies utilized immunodeficient mice, which do not fully recapitulate the complex tumor-immune interactions in human patients. Validation in immunocompetent models or patient-derived organoids would strengthen the translational relevance of our findings. Fourth, the clinical correlation between HSPA8-PPP2R2D axis activation and patient outcomes, particularly response to immunotherapy, requires validation in larger independent cohorts.

Fifth, we acknowledge that our current immune-related evidence is based on computational correlations (TIMER/CIBERSORT) and CD86/CD206 immunohistochemistry in xenograft tumors. These experiments demonstrate an association between tumor cell manipulation and changes in macrophage marker abundance, but do not directly demonstrate active macrophage polarization induced by the HSPA8-PPP2R2D-2 axis. Future studies employing *in vitro* conditioned media experiments with multiple M1/M2 markers are necessary to definitively establish direct tumor-to-macrophage crosstalk.

Sixth, we acknowledge that our *in vivo* studies were performed in BALB/c nude mice, which retain components of innate immunity but have profound T-cell deficiency. This substantially limits the interpretation of broad tumor immune microenvironment remodeling, as well as claims concerning “immune evasion” or implications for immune checkpoint therapy. Therefore, the macrophage-associated changes observed in this model should be clearly distinguished from the broader remodeling of an immunocompetent tumor immune microenvironment. Future studies using immunocompetent syngeneic mouse models are warranted to validate these findings.

Finally, a compelling future direction is the potential interaction between HSPA8 and other HSP70 family members or co-chaperones in the context of splicing regulation. HSP70 molecular chaperones are known to cooperate in supporting client protein function under stress conditions (21). Whether HSPA8 forms heterocomplexes with other chaperones in the nucleus to regulate splicing—potentially through interactions with spliceosomal components—remains an open and intriguing question. Investigating this possibility could reveal a broader chaperone network that governs both proteostasis and RNA processing in cancer cells.

## 5 Conclusion

In conclusion, this study identifies HSPA8 as a critical splicing modulator for PPP2R2D, likely through its association within the splicing machinery, to drive TNBC progression through dual mechanisms: direct suppression of mitochondrial apoptosis and changes in the tumor immune microenvironment associated with shifts in macrophage marker abundance. These findings reveal a previously unrecognized layer of TNBC biology and identify the HSPA8-PPP2R2D-2 axis as a potential therapeutic target for this aggressive malignancy.

## Data Availability

The raw sequence data reported in this paper have been deposited in the Genome Sequence Archive (Genomics, Proteomics & Bioinformatics 2025) in National Genomics Data Center (Nucleic Acids Res 2025), China National Center for Bioinformation / Beijing Institute of Genomics, Chinese Academy of Sciences (GSA-Human: HRA020727) that are publicly accessible at https://ngdc.cncb.ac.cn/gsa-human.

## Glossary

ANOVA: Analysis of variance
AS: Alternative splicing
BCA: Bicinchoninic acid
CCK-8: Cell Counting Kit-8
cDNA: Complementary DNA
CIBERSORT: Cell-type Identification By Estimating Relative Subsets Of RNA Transcripts
DAB: 3,3’-Diaminobenzidine
DEGs: Differentially expressed genes
DMEM: Dulbecco’s Modified Eagle Medium
ECL: Enhanced chemiluminescence
EDTA: Ethylenediaminetetraacetic acid
FBS: Fetal bovine serum
FC: Fold change
FDR: False discovery rate
FITC: Fluorescein isothiocyanate
GAPDH: Glyceraldehyde-3-phosphate dehydrogenase
GEPIA: Gene Expression Profiling Interactive Analysis
H&E: Hematoxylin and eosin
HER2: Human epidermal growth factor receptor 2
HRP: Horseradish peroxidase
HSPA8: Heat shock protein family A (Hsp70) member 8
IGV: Integrative Genomics Viewer
IHC: Immunohistochemistry
KEGG: Kyoto Encyclopedia of Genes and Genomes
PAGE: Polyacrylamide gel electrophoresis
PBS: Phosphate-buffered saline
PCR: Polymerase chain reaction
PI: Propidium iodide
PP2A: Protein phosphatase 2A
PPP2R2D: Protein phosphatase 2 regulatory subunit B delta
PSI: Percent spliced in
PVDF: Polyvinylidene fluoride
qPCR: Quantitative polymerase chain reaction
RIP: RNA immunoprecipitation
RNA-seq: RNA sequencing
RT-PCR: Reverse transcription polymerase chain reaction
RT-qPCR: Reverse transcription quantitative polymerase chain reaction
SD: Standard deviation
SDS: Sodium dodecyl sulfate
shRNA: Short hairpin RNA
siRNA: Small interfering RNA
STR: Short tandem repeat
TAMs: Tumor-associated macrophages
TBST: Tris-buffered saline with Tween 20
TCGA: The Cancer Genome Atlas
TIMER: Tumor Immune Estimation Resource
TNBC: Triple-negative breast cancer
TSVdb: Tumor Splice Variant database
